# Screening immune adjuvants for an inactivated vaccine against *Erysipelothrix rhusiopathiae*

**DOI:** 10.3389/fvets.2022.922867

**Published:** 2022-07-26

**Authors:** Li-Jun Guan, Shi-Xuan Pei, Ji-Jian Song, Peng-Fei Zhan, Yi-Nong Han, Yun Xue, Ke Ding, Zhan-Qin Zhao

**Affiliations:** ^1^Lab of Veterinary Microbiology, College of Animal Science and Technology, Henan University of Science and Technology, Luoyang, China; ^2^College of Veterinary Medicine, Jilin University, Changchun, China

**Keywords:** *Erysipelothrix rhusiopathiae*, swine, vaccine, adjuvant, protective efficacy

## Abstract

In this study, we screened adjuvants for an inactivated vaccine against *Erysipelothrix rhusiopathiae* (*E. rhusiopathiae*). Inactivated cells of *E. rhusiopathiae* strain HG-1 were prepared as the antigen in five adjuvanted inactivated vaccines, including a mineral-oil-adjuvanted vaccine (Oli vaccine), aluminum-hydroxide-gel-adjuvanted vaccine (Alh vaccine), ISA201-biphasic-oil-emulsion-adjuvanted vaccine (ISA201 vaccine), GEL02-water-soluble-polymer-adjuvanted vaccine (GEL vaccine), and IMS1313-water-soluble-nanoparticle-adjuvanted vaccine (IMS1313 vaccine). The safety test results of subcutaneous inoculation in mice showed that Oli vaccine had the most severe side effects, with a combined score of 35, followed by the ISA201 vaccine (25 points), Alh vaccine (20 points), GEL vaccine (10 points), and IMS1313 vaccine (10 points). A dose of 1.5LD_50_ of strain HG-1 was used to challenge the mice intraperitoneally, 14 days after their second immunization. The protective efficacy of Oli vaccine and Alh vaccine was 100% (8/8), whereas that of the other three adjuvanted vaccines was 88% (7/8). Challenge with 2.5LD_50_ of strain HG-1 resulted in a 100% survival rate, demonstrating the 100% protective efficacy of the Oli vaccine, followed by the GEL vaccine (71%, 5/7), IMS1313 vaccine (57%, 4/7), ISA201 vaccine (43%, 3/7), and Alh vaccine (29%, 2/7). Challenge with 4LD_50_ of strain HG-1 showed 100% (7/7) protective efficacy of the Oli vaccine and 71% (5/7) protective efficacy of the GEL vaccine, whereas the protective efficacy of other three adjuvanted vaccine was 14% (1/7). The Alh and GEL vaccines were selected for comparative tests in piglets, and both caused minor side effects. A second immunization with these two adjuvanted vaccines conferred 60 and 100% protective efficacy, respectively, after the piglets were challenged via an ear vein with 8LD_100_ of strain HG-1. After challenge with 16LD_100_ of strain HG-1, the Alh and GEL vaccines showed 40% and 100% protective efficacy, respectively. Our results suggested that GEL is the optimal adjuvant for an inactivated vaccine against *E. rhusiopathiae*.

## Introduction

*E. rhusiopathiae* is a Gram-positive rod-shaped nonspore-forming bacterium with a capsule but no flagellum (1), belonging to the genus *Erysipelothrix* in the family Erysipelothrichaceae. The bacterium is widely distributed in nature, causing diseases in a wide range of animals, including mammals, birds and fish (2–5). Humans can be infected via trauma that results in skin lesions, thus acquiring erysipelas (6). In swine, *E. rhusiopathiae* causes swine erysipelas, an acute, febrile contagious disease. Its main clinical manifestations include acute sepsis, subacute exanthematous endocarditis, and chronic endocarditis (2, 7). Based on heat-stable cell wall antigen, *Erysipelothrix* spp. strains can be divided into at least 28 serotypes (types 1a, 1b, 2-26, and N) (2, 8, 9). Strains of *E. rhusiopathiae* were determined as belonging to serovars 1a, 1b, 2, 4–6, 8, 9, 11, 12, 15–17, 19, 21, 23, and N (7, 10). The serotypes display distinct virulence, and types 1a and 1b are more virulent than the other serotypes (2, 11). So far, types 1 and 2 are the most strongly epidemic serotypes in pigs (7, 10, 12–15).

In the 1980–1990s, erysipelas was widespread and caused great economic losses in the swine industries of North America, Europe, Asia, and Australia (2, 7). This disease was also one of the three major infectious diseases (swine fever, swine erysipelas, and swine pasteurellosis) that threatened the swine industry in China. However, after the introduction of antibiotics, vaccines, and large-scale farming, swine erysipelas was considered to have disappeared. However, since 2010, swine erysipelas outbreaks have occurred again in the United States, Japan, Brazil, China, and other countries (14–19). *E. rhusiopathiae* vaccines are generally considered effective in preventing erysipelas. Traditionally, inactivated and live attenuated vaccines of *E. rhusiopathiae* serovar 1a or 2 isolates are still used today on a global basis (10). Additionally, some researchers identified several key immunogenic surface proteins of *E. rhusiopathiae* include surface protective antigen A (SpaA) (20), choline binding protein B (CbpB), *rhusiopathiae* surface protein A (RspA), and Glyceraldehyde 3-phosphate dehydrogenase (GAPDH) (21). Among them, single SpaA protein can protect immunized animals against lethal dose challenge of *E. rhusiopathiae*, which has potential as a candidate protein of *E. rhusiopathiae* subunit vaccine (20–24). Commercial erysipelas bacterins are still widely used today, and the inactivated vaccine preparation technology is mature and low in cost.

To optimize the immune effects of an inactivated vaccine for erysipelas, the selection of the appropriate adjuvant is essential, which a good adjuvant enhances the immune response to the antigen, and thus the immune effects of the vaccine (25, 26). Aluminum hydroxide adjuvant and oil adjuvant, known as the conventional and commercial adjuvant, are widely used in veterinary vaccines globally (27, 28). In recent years, various new adjuvants have appeared, Montanide™ adjuvants are a well-established brand of vaccine adjuvants used in veterinary vaccines. Montanide™ ISA201 is a water-in-oil-in-water (W/O/W) mineral oil-based adjuvant emulsion inside (29). The main component is highly refined light mineral oil, and it also contains small amounts of plant-derived mannitol and oleic acid (30, 31). The vaccine prepared with this adjuvant had a different water/oil dosage form (W/O) from that of the Oli vaccine, which reduced the viscosity of the vaccine, improved its injectability, and reduced its side effects, while maintaining the antigen delivery capacity of a mineral oil adjuvant. Montanide™ IMS 1313 VG N is a ready-to-dilute adjuvants, consisting of a water-dispersed liquid nanoparticles combined with an immunostimulating compound. This adjuvant can induce a rapid immune response with a strong sustainability especially in case of two-shot vaccination regimen (32). Montanide™ GEL, a new adjuvant based on the dispersion of polymeric gel in water, has the advantages of a high antigen load, high stability, and easy emulsification (33–35). Due to polymer adsorption properties, this adjuvant improves the recruitment of the innate immune system, which provide a significant enhancement of the immune response in aqueous vaccines (36).

In previous research, we compared the efficacy of several novel adjuvants in the Gram-negative bacteria *Glaesserella parasuis* trivalent inactivated vaccine. In this study, the HG-1 strain of type 1a *E. rhusiopathiae* was isolated from diseased swine in Guangdong Province in June 2018, and we aimed to validate the efficacy of novel adjuvant ISA201, IMS1313 and GEL in the Gram-positive bacteria *E. rhusiopathiae* inactivated vaccine using the newly isolated strain against the current epidemic strain. In addition, two traditional adjuvants, aluminum gel and mineral oil, were used as controls to screen for the optimal inactivated vaccine for swine erysipelas.

## Methods

### Strains and animals

The HG-1 strain of type 1a *E. rhusiopathiae* was isolated from diseased swine in Guangdong Province by the Veterinary and Biological Products Engineering Laboratory of Henan University of Science and Technology (Luoyang, China) in June 2018. According to Reed-Muench method (37), the 50% lethal dose (LD_50_) of this strain in 10–12-week-old BALB/c mice was 218 colony-forming units (CFU). Meanwhile, piglets were challenged with different doses of HG-1 strain, the 100% lethal dose (LD_100_) in 8–9-week-old healthy susceptible piglets was 1.0 ×10^4^ CFU. Specific-pathogen-free female BALB/c mice (6–8 weeks old) were purchased from Charles River Inc. (Beijing, China), and 4–5-week-old healthy susceptible piglets were purchased from farmers in Yichuan County, Henan Province. Healthy susceptible piglets were defined as piglets from groups with no outbreak of erysipelas in the preceding 2 years, with no clinical symptoms of swine erysipelas, and that had not been vaccinated for swine erysipelas. The sera of the healthy piglets also tested negative for types 1 and 2 *E. rhusiopathiae* in an enzyme-linked immunosorbent assay (ELISA) (38). All animal experiments in this study were performed in strict accordance with the recommendations delineated in the guidelines of the Animal Care and Use Committee of Henan University of Science and Technology (No. 20200610002).

### Materials and reagents

Tryptic soy agar (TSA) and tryptic soy broth (TSB) were purchased from Becton Dickinson Inc. (USA). Mycoplasma-free newborn bovine calf serum was purchased from TianHang Biotechnology Co, Ltd (Zhejiang, China). Sodium chloride and other reagents were purchased from Yili Reagents Co., Ltd (Beijing, China). The mineral oil adjuvant Marcol-52 (Oli) was purchased from ExxonMobil Corp. (USA); the aluminum hydroxide gel adjuvant (Alh) was purchased from General Chemical Corp. (USA); Montanide™ ISA201 VG biphasic oil emulsion adjuvant (ISA201), Montanide™ GEL02 PR soluble polymer adjuvant (GEL), and Montanide™ IMS 1313 VG N soluble nanoparticles (IMS1313) were produced by Seppic (Paris, France). ELISA reagents were purchased from Sangon Biotech (Shanghai, China).

### Preparation of the vaccine

*E. rhusiopathiae* strain HG-1 was inoculated with TSA medium containing 10% newborn bovine serum and incubated at 37°C for 24–48 h. Single colonies were picked and cultured in TSB medium containing 10% newborn bovine serum. The TSB liquid medium was then shaken at 37°C at 200 r/min for 16 h, and then diluted 1:100 with TSB medium containing 10% newborn bovine serum and shaken for 16 h at 37°C. The numbers of *E. rhusiopathiae* cells were enumerated by plate counts at 12 h. Formaldehyde (0.3% v/v) was added to the bacterial suspension, which was inactivated at 37°C for 48 h. The fully inactivated bacterial suspension was centrifuged and the supernatant discarded, and resuspended with sterile phosphate-buffered saline (PBS, 0.01 mol/L, pH 7.2), that is, HG-1 inactivated antigen was obtained. The inactivated Oli and Alh vaccines were prepared with previously reported methods (33, 39), with a 2:1 ratio of oil adjuvant to HG-1 antigen in oil vaccine preparation, and a 1:4 ratio of Alh to HG-1 antigen in Alh vaccine. The ISA201 vaccine, IMS1313 vaccine, and GEL vaccine were prepared according to their respective adjuvant manufacturer's instructions. Briefly, HG-1 antigen and ISA201 were pre-warmed separately in a water bath (31°C) for 30 min, and then HG-1 antigen was dropped into ISA201 slowly to a final ratio of 1:1 (w/w), with gentle shaking, as ISA201 vaccine. IMS1313 was dropped into HG-1 antigen to a final ratio of 1:1 (v/v) slowly, with gentle shaking, as IMS1313 vaccine. GEL 02 was slowly dropped into HG-1 antigen at a final ratio of 1:4 (v/v) and gently shaken, as GEL vaccine. The formulations were incubated for 30 min at room temperature with constant shaking. The final killed whole-cell vaccine (3.0 × 10^9^ killed cells/mL) was stored at 4°C for further analysis.

### Safety test in mice

Thirty BALB/c mice were randomly divided into six groups (five mice per group), and the five adjuvanted vaccines (Oli vaccine, Alh vaccine, GEL vaccine, ISA201 vaccine, and IMS1313 vaccine) were used to vaccinate the five groups *via* subcutaneous injection on the back (0.2 mL per mouse). One control group of five mice was injected with sterile PBS (0.2 mL per mouse). The mice were observed and recorded for 14 days after vaccination. The safety assessment criteria include: 1. death; 2. clinical symptoms including depression, unkempt fur, appetite, vomiting, swelling at the injection site, and diarrhea, which lasts for at least 3 days; meanwhile, necropsied to check for inflammation, necrosis, and vaccine residue at the injection site. If none of the above symptoms occur, no points will be awarded. One point for mild symptoms; two points for moderate symptoms; three points for severe symptoms. A comprehensive score was calculated according to the intensity of the clinical symptoms and lesion indicators to determine the severity of the side effects in the vaccinated mice.

### Protective efficacy in vaccinated mice

One hundred thirty-two mice were randomly divided into three groups: 48 in group 1 and 42 each in groups 2 and 3. Each group was randomly divided into six subgroups: subgroups 1–5 were vaccinated subcutaneously with one of the five adjuvanted vaccines and subgroup 6 was injected with sterile PBS (0.2 mL per mouse) as the control. A second vaccination was administered 21 days after the primary vaccination. Blood samples were collected from the mouse tail veins and the serum separated before vaccination and after the primary and secondary vaccinations, and were used to the test antibody levels with an ELISA (23). Fourteen days after the secondary vaccination (35 days after the primary vaccination), all three groups of mice were injected intraperitoneally with 1.5LD_50_, 2.5LD_50_, or 4LD_50_ (LD_50_ is 218 CFU) of strain HG-1. Their clinical symptoms, morbidity, and death were observed and recorded for 14 days. The dead mice were necropsied and the bacteria were isolated and identified.

### Safety test in piglets

Based on the results of the test described in sections 2.4 and 2.5, the Alh vaccine and GEL vaccine for *E. rhusiopathiae* were selected for further safety testing in piglets. Fifteen piglets were randomly divided into three groups (five per group), and the piglets in groups 1 and 2 were vaccinated with double-strength Alh vaccine or GEL vaccine *via* an intramuscular injection in the neck (4 mL each). The blank control group was injected with sterile PBS (4 mL each). The clinical symptoms of the piglets, including their mental status, appetite, and death, were recorded for 14 days. The piglets in each group were necropsied to check for inflammation, necrosis, and vaccine residue at the injection site.

### Immune protection test in piglets

Thirty piglets were randomly divided into six groups (five per group). Groups 1 and 2 were injected in the neck with 2 mL of Alh vaccine; groups 3 and 4 were injected in the neck with 2 mL of GEL vaccine; groups 5 and 6 were injected with sterile PBS (2 mL) as the control groups. A second immunization was performed 21 days later with the same dose. Neck vein blood samples were collected from each piglet before and after the primary immunization and after the secondary immunization. The sera were separated from the blood samples to test their antibody levels with an ELISA. Fourteen days after the second immunization, 8LD_100_ (8.2 × 10^4^ CFU) of strain HG-1 (1.0 mL) was injected into the ear veins of the piglets in groups 1, 3, and 5, and 16LD_100_ (1.6 × 10^5^ CFU) of strain HG-1 (1.0 mL) was injected into the ear veins of the piglets in groups 2, 4, and 6. Morbidity and mortality were observed and recorded for 14 days. The sick judgment criteria are as follows: 1. death. 2. clinical symptoms including depression, lethargy, pyrexia, inappetence, dyspnea, joint swelling, lameness, lying down, and characteristic pink, red, or purple raised firm rhomboid or squared “diamond skin” lesions, which lasts for at least 3 days; meanwhile, autopsy showed systemic septicemia, including lymph nodes, spleen and lung enlargement and congested, petechiae and ecchymosis may be found in the renal cortex and heart (epicardium and atrial myocardium). In addition, serosanguinous effusion in the joint cavity is also observed, and the joint capsule is often hyperemic. The dead piglets were necropsied for bacterial identification.

### Data analysis

The data were analyzed with SPSS version 25.0. Serum antibody levels were analyzed with a *t-*test (Student's *t-*test) and one-way analysis of variance (ANOVA). Multiple comparisons were evaluated with Duncan's method. The protective efficacy of vaccine was tested by chi-square test. *P* < 0.05 was considered to indicate significance, and *P* < 0.01 to indicate extreme significance.

## Results

### Vaccine safety in mice

**Figure 1:**
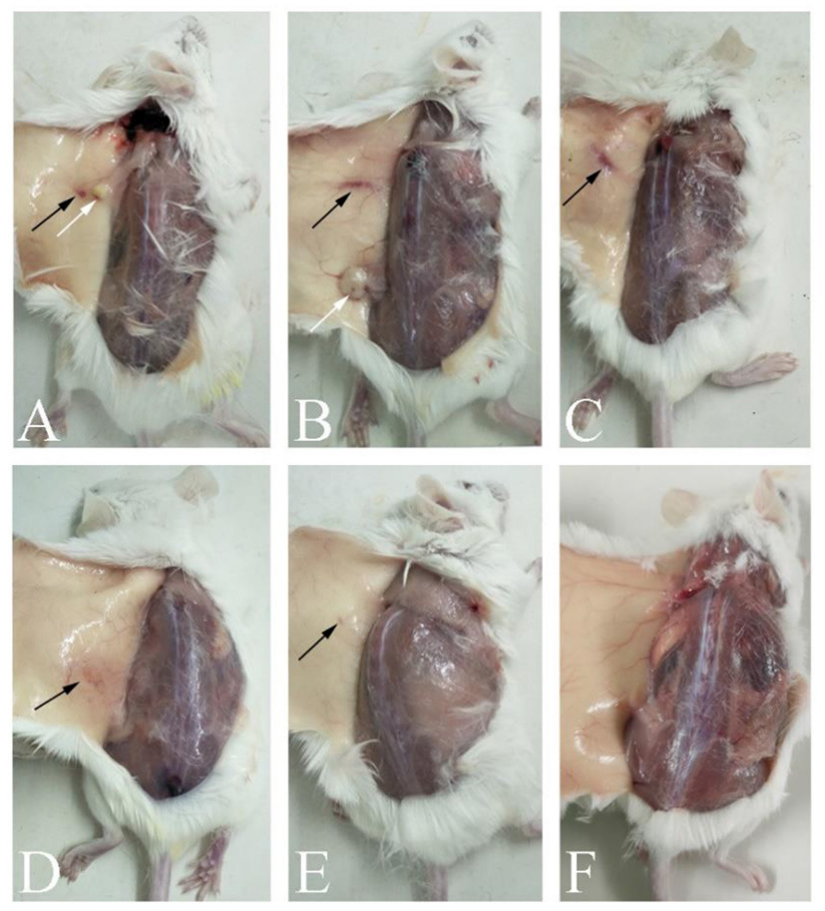
Autopsy changes in mice immunized with differently adjuvanted vaccines. **(A)** indicates the mice immunized with Oli vaccine; **(B)** indicates Alh vaccine; **(C)** indicates ISA201 vaccine; **(D)** indicates GEL vaccine, **(E)** indicates IMS1313 vaccine; and **(F)** indicates PBS control group. The black arrows in the figure showed foci of inflammation, and white arrows showed an inflamed, fluid-filled cyst.

We recorded the mental state, appetite, vomiting, diarrhea, and death in mice during the safety test (Table 1), and any inflammation, necrosis, or vaccine residue at the injection site (Figure 1). The results showed that the Oli vaccine caused serious side effects in mice, including messy fur and swelling and necrosis at the injection site, whereas the ISA201 and Alh vaccines caused less-serious side effects. ISA201 mainly caused local irritation and therefore messy hair, and the Alh vaccine mainly caused nodules and vaccine residue. The GEL and IMS1313 vaccines caused only minor side effects.

**Table 1:** Safety assessment of the vaccine with five different adjuvants in mice.

**Vaccine type**	**Number**	**Depression**	**Anorexia**	**Diarrhea**	**Emesis**	**Unkempt skin/fur**	**Necrosis**	**Vaccine residue**	**Death**	**Score**
Oli vaccine	1	+	-	-	-	+++	+	++	-	35
	2	+	-	-	-	+++	+	++	-	
	3	+	-	-	-	+++	+	++	-	
	4	+	-	-	-	+++	+	++	-	
	5	+	-	-	-	+++	+	++	-	
Alh vaccine	1	-	-	-	-	+	+	++	-	20
	2	-	-	-	-	+	+	++	-	
	3	-	-	-	-	+	+	++	-	
	4	-	-	-	-	+	+	++	-	
	5	-	-	-	-	+	+	++	-	
ISA201 vaccine	1	+	-	-	-	++	+	+	-	25
	2	+	-	-	-	++	+	+	-	
	3	+	-	-	-	++	+	+	-	
	4	+	-	-	-	++	+	+	-	
	5	+	-	-	-	++	+	+	-	
GEL vaccine	1	-	-	-	-	+	+	-	-	10
	2	-	-	-	-	+	+	-	-	
	3	-	-	-	-	+	+	-	-	
	4	-	-	-	-	+	+	-	-	
	5	-	-	-	-	+	+	-	-	
IMS1313vaccine	1	+	-	-	-	+	-	-	-	10
	2	+	-	-	-	+	-	-	-	
	3	+	-	-	-	+	-	-	-	
	4	+	-	-	-	+	-	-	-	
	5	+	-	-	-	+	-	-	-	
PBS control	1	-	-	-	-	-	-	-	-	0
	2	-	-	-	-	-	-	-	-	
	3	-	-	-	-	-	-	-	-	
	4	-	-	-	-	-	-	-	-	
	5	-	-	-	-	-	-	-	-	

Normal; +: slight; ++: moderate; +++: severe. One “+” is scored as one point, and the overall score indicates the severity of the vaccination response in mice.

### Detection of serum antibodies in immunized mice

**Figure 2:**
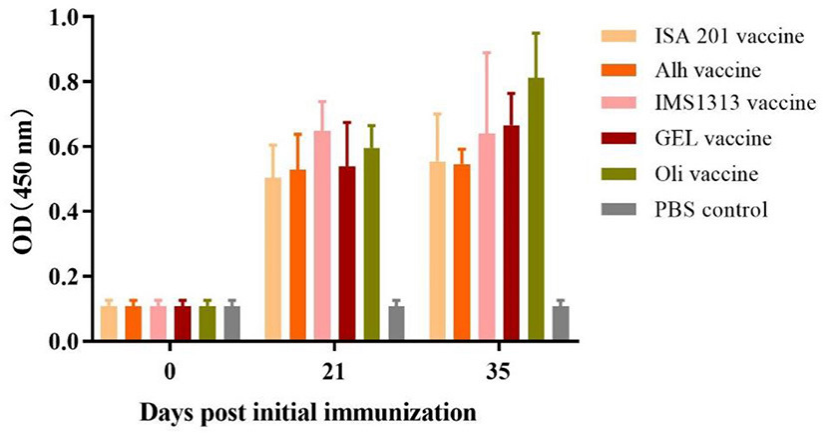
Antibody titers in mice immunized with the five differently adjuvanted vaccines. Blood samples were collected before vaccination and after the primary and secondary vaccinations. “ISA201 vaccine”, “Alh vaccine”, “IMS1313 vaccine”, “GEL vaccine” and “Oli vaccine” indicate that mice were subcutaneously immunized with the corresponding vaccine, respectively. “PBS control” indicates mice inoculated with sterile PBS buffer. The *E. rhusiopathiae*-specific antibody titers in the experimental and control groups were measured with an indirect ELISA and recorded at OD450. The error bars represent the SD.

The ELISA results for the immunized mice (Figure 2) showed that, 21 days after the first immunization, the serum antibody levels of mice vaccinated with the ISA201 vaccine, Alh vaccine, GEL vaccine, IMS1313 vaccine, or Oli vaccine were extremely significantly higher than that before immunization and control group (*P* < 0.01). At 14 days after the secondary immunization, the antibody levels in the mice of the Oli vaccine group were significantly increased (*P* < 0.05), whereas those in the mice of the other four groups were not (*P* > 0.05), compared with that of the corresponding immunization group after the first immunization. The serum antibody level of Oli vaccine group was significantly higher than that of Alh vaccine group or ISA201 vaccine group (*P* < 0.05), but there were no significant differences among other groups (*P* > 0.05). These results indicated that all five adjuvanted vaccines induced high levels of antibodies in mice. However, after the second immunization, none of the adjuvanted vaccines, except the Oli vaccine, resulted in significantly increased antibody levels.

### Protective efficacy in immunized mice

Mice were challenged with strain HG-1 at three different doses, 1.5LD_50_, 2.5LD_50_ and 4LD_50_, 14 days after the boost vaccination (Table 2). With the increase of challenge dose, the protective effect of Alh, ISA201 and IMS1313 vaccine groups declined, particularly when the challenge dose of strain HG-1 was 4LD50, the protective rate of the three vaccines was only 14% (*P* > 0.05) compared with the control group. However, compared with the corresponding control group at the same challenge dose, the protective rates of Oil (*P* < 0.05) and GEL vaccine (*P* < 0.05) groups were 100% when challenged with 1.5LD50 and 2.5LD50, and 100% and 71% when challenged with 4LD50, respectively. In addition, there was no difference in the protection rate between the Oil group and GEL group at any same challenge dose (Table 2). Autopsy of dead mice showed the typical pathological changes associated with sepsis.

**Table 2:** Protective efficacy of the vaccine with five different adjuvants in mice.

**Vaccine type**	**Vaccine dose (mL)**	**Challenge strain**	**Survive/Total**		
			**354 CFU**	**530 CFU**	**876 CFU**
Oli vaccine	0.2	HG-1	8/8^a^	7/7^a^	7/7^a^
Alh vaccine	0.2	HG-1	8/8^a^	5/7^a^	1/7^b^
GEL vaccine	0.2	HG-1	8/8^a^	7/7^a^	5/7^a^
ISA201 vaccine	0.2	HG-1	8/8^a^	6/7^a^	1/7^b^
IMS1313 vaccine	0.2	HG-1	7/8^a^	3/7^ab^	1/7^b^
PBS control	0.2	HG-1	2/8^b^	0^b^	0/7^b^

Comparison of the difference of protective effect of different immune groups at the same challenge dose, the same letter in the upper right corner of the score indicated that there is no difference in the protection rate between groups (P > 0.05), and different letters indicated significant difference, P < 0.05.

### Safety of vaccines in piglets

**Figure 3:**
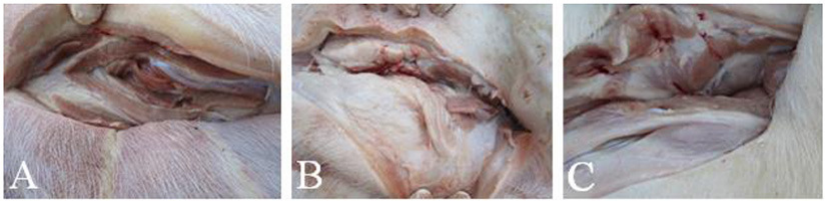
Vaccine residues in piglets immunized with differently adjuvanted vaccine. **(A)** represents GEL vaccine; **(B)** represents Alh vaccine; **(C)** represents PBS control group. After the observation period, the piglets were dissected to observe the intramuscular injection site of neck, and there was no visible difference between the two vaccine groups and the control group.

Piglets were vaccinated against *E. rhusiopathiae* with an intramuscular injection in the neck of the double-strength Alh vaccine or double-strength GEL vaccine (4 mL). There were no notable changes in the mental state or appetite of the piglets vaccinated with either vaccine. There were no marked differences in diarrhea, vomiting, or death between the vaccinated groups and the PBS-injected control group. The vaccinated piglets showed a transient increase in body temperature, but the average temperature increase did not exceed 1°C at 24 h after vaccination, and returned to normal within 48 h. Observation of the injection sites on the piglets showed no obvious local inflammation, tissue lesions, vaccine residue, or granulomas in the piglets vaccinated with either vaccine. Autopsies showed no notable differences between the vaccinated and the control groups (Figure 3). These results demonstrated the adequate safety of the two vaccines for piglets.

### Serum antibodies in immunized piglets

**Figure 4:**
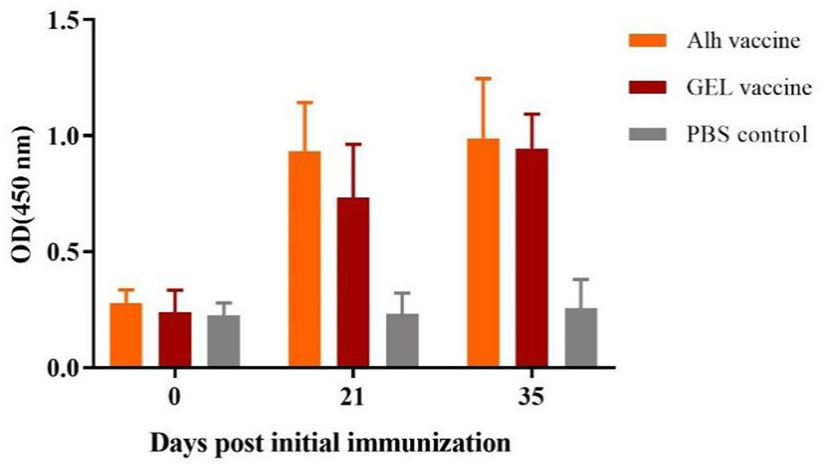
Antibody titers of piglets immunized with the two adjuvanted vaccines. Blood samples were collected before each immunization and before challenge. “Alh vaccine”, “GEL vaccine” and “PBS control” respectively indicate that piglets were subcutaneously immunized with the corresponding vaccine. The *E. rhusiopathiae*-specific antibody titers in the experimental and control groups were measured with an indirect ELISA and recorded at OD450. The error bars represent the SD.

The ELISA results for the immunized piglets (Figure 4) showed that, 21 days after the primary immunization, the serum antibody levels in the Alh-vaccinated and GEL-vaccinated groups were extremely significantly higher than that before immunization and control group (*P* < 0.01). However, 14 days after the secondary immunization, the serum antibody levels were not significantly higher than those after the primary vaccination (*P* > 0.05). There was also no significant difference between the two immunized groups (*P* > 0.05). This indicated that the two adjuvanted vaccines induced increased antibody levels after immunization, but no significant increase was observed after the second immunization.

### Immunization protects piglets

After the primary and secondary immunization of piglets with the GEL or Alh vaccine, we used 8LD_100_ or 16LD_100_ of the highly toxic HG-1 strain to challenge them *via* an ear vein injection (Table 3). After the 8LD_100_ challenge, the piglets in the control group showed disease within 48 h, which manifested as clinical symptoms including depression, loss of appetite, joint swelling, lameness and mucosal cyanosis. Three deaths occurred within 4 days of challenge. All piglets in GEL and Alh vaccine groups survived, but two piglets in Alh vaccine group were seriously ill (Table 3). After challenge with 16LD_100_, all piglets in the control group had succumbed to disease by 36 h after challenge, and all died within 4 days of challenge. Three piglets in Alh vaccine group showed clear clinical symptoms, and two were dead within 14 days (Table 3). Therefore, the protection rate of the Alh vaccine group was 60%. All the piglets in GEL vaccine group survived, and only two showed transient depression and loss of appetite, and fully recovered within 24 h. Therefore, the protection rate of the GEL vaccine was 100%. Autopsies were performed immediately on the dead piglets, and all showed typical septic pathological changes. *E. rhusiopathiae* was isolated from the organs and tissues of the dead piglets, including the heart, liver, spleen, lung, kidney, brain, lymph nodes, large intestine, and small intestine.

**Table 3:** Protective efficacy of the vaccine with two different adjuvants in piglets.

**Vaccine type**	**Vaccine dose (mL)**	**Challenge strain**	**Sick/Dead/Total**	
			**8.2** × **10**^4^ **CFU**	**1.6** × **10**^5^ **CFU**
Alh vaccine	2	HG-1	2/0/5	2/2/5
GEL vaccine	2	HG-1	0/0/5	0/0/5
PBS control	2	HG-1	5/3/5	5/5/5

## Discussion

Vaccination is the key to preventing swine erysipelas. For bacterins various adjuvant are added to provide longer immunity. Aluminum hydroxide, mineral oil, ISA201, GEL, and IMS1313 adjuvants, low-cost and enhancement of immune response, have been widely used in animal vaccine research. According to adjuvant manufacturer's instructions, the cost of different adjuvants from low to high is mineral oil which is 0.067 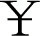/mL using 66.7% adjuvant in bacterin preparation, Alh adjuvant which is 0.09 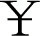/mL using 20% in bacterin preparation, ISA201 adjuvant (0.09 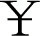/mL, 50%), IMS1313 adjuvant (0.25 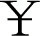/mL, 50%), and GEL adjuvant (0.26 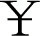/mL, 20% adjuvant for vaccine preparation), respectively. Traditional adjuvants have advantages in vaccine preparation costs, but the cost of novel adjuvants is acceptable. The safety and efficacy of vaccines are important indicators of their quality, and appropriate adjuvants are critical to the safety and efficacy of swine erysipelas vaccines.

Our study has shown that the Oli-adjuvanted vaccine induced the highest antibody titers in immunized mice and afforded complete (100%) protection against *E. rhusiopathiae*. However, this adjuvant also caused serious side effects, including messy fur, swelling, necrosis, and vaccine residue at the injection site (Table 1). This is consistent with the side effects observed in piglets immunized with the Oli vaccine, which included loss of appetite, slow growth, inflammation and necrosis at injection site, and granuloma (33, 40). Mineral oil adjuvants are gradually being replaced by a variety of other novel adjuvants in biological products for swine.

The ISA201 adjuvant is a new type of water/oil/water (W/O/W) dual-phase oil emulsion adjuvant, which has recently been widely used in a variety of animal vaccines (41–43). However, in this study, although ISA201 showed better safety in mice than traditional mineral oil adjuvants, its immune efficacy was significantly lower. The IMS1313 adjuvant is a water-soluble nanoadjuvant developed in recent years. Vaccines prepared with this adjuvant provided good protection in the low dose challenge experiment, but poor immunization efficacy in the high dose challenge experiment.

GEL is a new type of water-soluble polymer adjuvant, the main component of which is polyethylene acrylic acid sodium. The excellent effects of the GEL adjuvant have been verified in vaccines against several viruses, including an inactivated *Porcine circovirus* vaccine (44), an inactivated *Porcine reproductive and respiratory syndrome virus* vaccine (35), an inactivated equine influenza virus vaccine (45), a live attenuated porcine epidemic encephalitis vaccine (34), and a *Bovine herpesvirus* DNA vaccine (46). However, this adjuvant showed a poor adjuvant effect in an inactivated sheep paratuberculosis vaccine (30), a *Rhodococcusequi* subunit vaccine (47), and a bovine mite disease subunit vaccine (48). There have been few reports of GEL-adjuvanted bacterial vaccines. In our previous study of adjuvants for an *G. parasuis* vaccine, the GEL adjuvant showed markedly better safety than a mineral oil adjuvant and 100% protective efficacy, and was also more efficacious than other adjuvants, including aluminum gel, IMS1313, and ISA760 (33, 49, 50).

In this study, we compared the effects of different adjuvants on an inactivated vaccine against Gram-positive *E. rhusiopathiae* in swine. In tests on mice, the GEL adjuvant was safe, induced high antibody levels and provided better protection than the conventional aluminum hydroxide adjuvants and the novel ISA201 and IMS1313 adjuvants. Because an aluminum gel adjuvant was used with most conventional inactivated swine erysipelas vaccines, we also compared the GEL adjuvant with the Alh adjuvant in piglets. Our results showed the excellent safety of both adjuvants, but the GEL adjuvant conferred greater protection (100%). The Alh vaccine showed a 60% protection rate. The antibody levels induced by the Alh vaccine and GEL vaccine after the second immunization were similar, but conferred different rates of protection in the challenge experiment. This may be attributable to we only measured the total IgG antibody levels in this study, but the aluminum gel adjuvant mainly stimulates the Th2 reaction and thus the humoral immune response, which the corresponding antibodies produced are mostly IgG1 (28, 51, 52). The GEL adjuvant is a new polymer adjuvant that has been used in recent years. Recent studies have shown that it not only induces humoral immunity, but also significantly promotes T-lymphocyte proliferation and differentiation, resulting in high-level cellular immunity (34, 44). Therefore, the two vaccines may cause different immune responses. In addition, it is puzzling that after booster immunization, the serum antibody levels of piglets and mice (except Oli vaccine) immunized group did not increase significantly compared with that after the first immunization, which needs further analysis.

Our results indicated that the GEL adjuvant not only showed great efficacy and a higher protection rate when used in an inactivated vaccine for Gram-negative *G. parasuis* (33), but also has great potential utility in inactivated bacterial vaccines against Gram-positive *E. rhusiopathiae*.

## Conclusion

In this study, we used the type 1a HG-1 strain of *E. rhusiopathiae* as the antigen to construct an inactivated vaccine against erysipelas with five different adjuvants, and tested the safety and immunization efficacy of the five differently adjuvanted vaccines in mice. This preliminary evaluation suggested that the GEL adjuvant was an excellent adjuvant, with good immunoprotective efficacy. When the GEL vaccine and the conventional Alh vaccine were used to immunize piglets, the GEL vaccine exerted fewer side effects and conferred markedly better immune protection than the Alh vaccine. This study provides strong evidence upon which to base the development of new inactivated vaccines against swine erysipelas.

## Data availability statement

The original contributions presented in the study are included in the article/supplementary material, further inquiries can be directed to the corresponding author.

## Ethics statement

All procedures performed in studies involving animals were approved by the Animal Care and Use Committee of Henan University of Science and Technology (No. 20200610002).

## Author contributions

L-JG, S-XP, and Z-QZ conceived and designed the study. L-JG and S-XP performed the experiments. J-JS, P-FZ, and Y-NH analyzed the data. L-JG, S-XP, YX, KD, and Z-QZ wrote and revised the manuscript. All authors read and approved the final version.

## Funding

This work was supported by grants from the National Natural Science Foundation of China (U1704117 and 32072899).

## Conflict of interest

The authors declare that the research was conducted in the absence of any commercial or financial relationships that could be construed as a potential conflict of interest.

## Publisher's note

All claims expressed in this article are solely those of the authors and do not necessarily represent those of their affiliated organizations, or those of the publisher, the editors and the reviewers. Any product that may be evaluated in this article, or claim that may be made by its manufacturer, is not guaranteed or endorsed by the publisher.
